# An Update on Syndemics: Editorial Comments

**DOI:** 10.3390/tropicalmed10070187

**Published:** 2025-07-02

**Authors:** Nicola Bulled, Merrill Singer

**Affiliations:** Institute for Collaboration on Health, Intervention, and Policy (InCHIP), University of Connecticut, 2006 Hillside Road, Storrs, CT 06269, USA

The theory of syndemics hypothesizes that observed clusters of diseases in specific temporal and geographical contexts are the result of harmful socio-environmental conditions resulting in mutually enhancing deleterious consequences. For the past 30 years, the concept has informed an array of health-related disciplines, proving valuable in health research, policy, practice, and education. It has been used to guide recent responses to MPOX [1], COVID-19 [2], and Ebola [3].

For this Special Issue, scholars working in the field were asked to consider novel syndemic attributes of clusters of infectious diseases that result in worse health outcomes than each condition in isolation. While HIV-related syndemics remain the most studied, new disease arrangements have emerged that warrant investigation including those related to novel infectious disease epidemics and re-emerging epidemics, as well as chronic epidemics that, historically, have received limited attention and resources. 

In keeping with the three tenets of syndemics, scholars were asked to consider the following: (1) clusters of infectious diseases and other health conditions concentrated in a population; (2) the biological interactions of diseases in a manner that increases infectivity or disease progression beyond that of co-existing/comorbid disease arrangements; and (3) the social, structural, and/or environmental conditions that support the existence of multiple health conditions within a population.

**Tenet 1: Clustering of epidemic diseases in a population.** Multiple morbidities or comorbidities, the co-occurrence of multiple diseases or health conditions, is well established in the literature. Certain conditions, such as HIV, given its impact on the body’s immune response and promoting inflammation, are well recognized as being associated with a host of varied comorbidities including other communicable diseases, chronic non-communicable diseases, and mental health conditions. 

While the clustering of health conditions may be seen as serially causal or sequential, with one condition preceding the other, disease interactions are often more dynamic and complex and influenced by the social context. For example, untreated syphilis can facilitate the transmission and acquisition of HIV infection [4]. In turn, HIV increases risk of syphilis acquisition [5] and can alter syphilis manifestations, potentially misleading clinical diagnosis [4]. Co-infection can result in a higher risk of treatment failure, and genital ulcers take longer to heal in patients with HIV than in patients with syphilis alone, increasing the risk of exposure to other sexually transmitted infections [4,5]. These conditions are understood to share “common risk factors”, indicating that they cluster among individuals who engage in risk behaviors, hold specific social identities, and experience structural risk factors. 

All eight of the articles in this Special Issue highlight the clustering of diseases within a defined population. McCollum et al. [6] present a novel arrangement of Neglected Tropical Diseases (NTDs) of the skin and mental distress in Liberia. Using a mixed-methods approach to understanding the experiences of individuals with NTDs of the skin, the authors found that disability was significantly associated with higher levels of depression and anxiety, with persons affected experiencing additional financial concerns, stigma, and pain, which all contributed to their mental health conditions. Barret et al. [7] also consider the mental health burden of infectious diseases as they detail syndemics of Lymphatic Filariasis in Malawi. For people affected by Lymphatic Filariasis, absent medical referral pathways, inequalities in healthcare provision or available treatment, and limited knowledge of the condition heightened patients’ mental distress. Distress was further exacerbated by stigma and social exclusion, and was shaped by the intersections of gender, poverty, and extreme climate conditions. Collectively, the articles in this Special Issue reveal the value of the syndemics framework in both recognizing previously unexplored comorbidities and considering disease clustering within populations experiencing specific shared social conditions, to focus and mobilize public health and healthcare resources.

**Tenet 2: Detailing the interaction of diseases.** Disease interaction is a key unique feature of syndemic theory, distinguishing it from the existing frameworks of *comorbidities* and *social determinants of health*. Syndemic theory recognizes that clustered and comorbid diseases interact in a manner that exacerbates the health outcomes of diseases beyond what would be expected from the combination of their independent effects. The name *syn*-demic implies that this interaction is *syn*-ergistic, where the combined effect is greater than the sum of the separate effects. 

Presently, the syndemics literature is struggling to address this second tenet. Syndemic theory has been criticized for its inability to clearly articulate and present empirical measures of synergistic interaction [8,9]. The criticism brings to light many questions. How do we present evidence of interaction? What counts as evidence of interaction? Do we have statistical methods or analyses to test hypotheses of interactions of multiple conditions simultaneously? 

On the one hand, the criticism suggests that the theory has yet to be evidenced; on the other hand, it raises a larger question about what counts as evidence. Statistically measuring disease interactions does not provide details on the nature of the intricate and complex responses of the body’s immune system. Furthermore, current statistical tools only allow us to measure the interaction between two co-existing phenomena, limiting the examination of disease clusters that involve more than two comorbidities. Disease interaction not only still exists but also still matters, irrespective of our ability to measure it [10]. Disease interactions can and have been demonstrated by combining evidence from various forms including literature on disease physiology, epidemiology, cohort studies, clinical trials, and ethnographies or qualitative studies detailing experiences of disease. These assessments present clear evidence supporting syndemic theory.

However, the complexity in fully articulating interaction in syndemic structures is evident in the scoping review of the cardiovascular disease syndemic literature conducted by Quiroz-Mena and colleagues [11] in this Special Issue. The results showed that few studies are adherent to the elements of syndemic theory. The dominant quantitative method to provide empirical evidence supporting disease interaction remains the “sum scores” or cumulative disease approach. The approach assesses whether higher disease concentration is associated with more severe health outcomes; it does not consider the nature of the disease interactions, but does provide evidence of clustering [8,9,12,13]. 

Tripp et al. [14] present a historical case of the 1918/19 influenza pandemic, using life table analysis to operationalize the syndemic of influenza and pulmonary tuberculosis among impoverished and marginalized adults of reproductive age (20–34 years) in Malta. They show that individuals of reproductive age had a significant increase in pulmonary tuberculosis mortality during the 1918 influenza period, and that those with both infections had a higher probability of mortality than those with only one infection. This does not prove disease interaction, indicating only the accumulation of disease risk. However, there is substantial evidence of biological interaction between the diseases, and the authors point to other studies that have similar findings of higher rates of mortality in individuals infected with both influenza and pulmonary tuberculosis. Tripp et al. complete their presentation of the syndemic of pandemic influenza and pulmonary tuberculosis in Malta by considering the impact of socioeconomic and environmental factors such as the increased cost of living, increased unemployment, political upheaval, temperature, and relative humidity. 

Stopka et al. [15] presented a robust empirical analysis of the clustering of serious bacterial infections and Hepatitis C (HCV). Of the participants reporting a past-year hospitalization for a serious bacterial infection, most tested HCV-antibody-positive. To account for disease interaction, the prevalence ratios of past-year serious bacterial infections were calculated with each risk factor in separate models, and the effect of modifications was assessed using multiplicative interaction. No evidence of interaction between HCV and serious bacterial infections was found. Their results indicate that the co-occurrence of HCV and serious bacterial infection is likely mediated by shared risk behaviors. Although no evidence of disease interaction was found in this study population, this is likely due to the small sample size. It is well established that HCV-related liver damage impairs the body’s ability to clear bacterial infections, increasing disease susceptibility and severity. This paper indicates how challenging it is to prove disease interaction using present statistical techniques.

**Tenet 3: Social vulnerabilities driving disease clusters.** Syndemics theory recognizes that disease clusters tend to appear in socially vulnerable populations, indicating that the social, structural, and environmental conditions shared by a population drive disease risk. Furthermore, while epidemics may transcend national borders, driven by various influencing social factors, syndemic diseases take on unique arrangements in different social, political, economic, cultural, and geographic contexts [16,17]. As such, place-based assessments are much needed to develop context-specific solutions. 

All of the papers published in this Special Issue attest to the multitude of interacting and intersecting social vulnerabilities that support the existence of clustering disease epidemics within a population. For example, the review by Hernandez Barrios et al. [18] focused on the issue of population vulnerability in the context of the COVID-19 pandemic. Their analysis of 40 articles published between December 2019 and October 2022 found that vulnerability is a systemic issue, with COVID-19 control measures directly increasing vulnerability in some populations. Villacis-Alvarez and colleagues [19] examined gendered and intersecting barriers and facilitators across the range of HIV care before and during the COVID-19 pandemic, which included COVID-19-associated disruptions in services and support structures. This compounded mental health challenges, substance use, violence (including intimate partner violence), internalized and enacted compounded stigma, and discrimination among individuals with HIV. 

Bulled [20] contributes to the burgeoning literature on occupational syndemics, detailing the conditions of wine farmworkers in the Western Cape, South Africa. These include dangerous workplace conditions, substandard housing, limited access to healthcare, labor policies that fail to protect workers and worker rights, limited state oversight, and historical legacies of the “dop” (tot) system of payment through alcohol. The occupational and structural factors experienced by farmworkers in the Cape Winelands result in high levels of interacting diseases including HIV, tuberculosis, metabolic syndrome, problem alcohol use, and interpersonal violence.

This confluence of social and structural factors is often difficult to isolate, measure, dissect, and account for causation. Consequently, the elements that drive disease clusters are often disregarded or not fully explored. What is more concerning is that, given new guidance for research studies funded by the US government, future studies may not be allowed to address issues of inequality/inequity, marginalization, and disparity. As such, this third tenet of syndemic theory, which recognizes that those made vulnerable to disease clusters due to historic and present policies, geographies, environments, economies, and culture, may, in the future, be left unexamined. The result will be that either disease burdens ravaging select communities may be unidentified, or significant amounts of resources will be wasted on entire populations to address overlapping disease epidemics that primarily affect communities with unique biological and social characteristics.

The set of papers in this Special Issue adds to the significant body of research and review on syndemics and attests to the value of syndemics theory across health-related disciplines. Future work on syndemics and infectious disease will need to adhere to robust analytic methods for qualitative, quantitative, and mixed-method studies of syndemic interaction and the translation of research into effective intervention. Such research may face increasing challenges based on ideological grounds, a turn of events that will hinder future progress on syndemics and limit its contributions to public health.
